# Supplementary material for: Solid-State Li-Ion Batteries Operating at Room Temperature Using New Borohydride Argyrodite Electrolytes
Source: Materials (Basel). 2020 Sep 11;13(18):4028. doi: 10.3390/ma13184028 (PMC7558157; doi:10.3390/ma13184028)
Supplement: Supplementary file 1 [file materials-13-04028-s001.pdf]

materials

Supplementary Materials

Solid-State Li-ion Batteries Operating at Room Temperature Using New Borohydride Argyrodite Electrolytes.

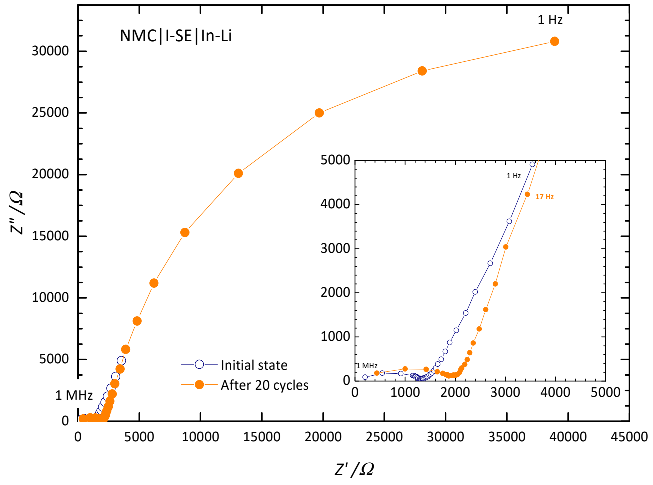

Figure S1: Impedance profiles of the NMC|I-SE|In-Li cell obtained before (open purple circle) and after (full orange circle) 20 cycles at 25 °C.

© 2020 by the authors. Submitted for possible open access publication under the terms and conditions of the Creative Commons Attribution (CC BY) license (http://creativecommons.org/licenses/by/4.0/).
